# Electrical Anaesthesia

**Published:** 1859-02

**Authors:** W. G. Oliver

**Affiliations:** Buffalo, N. Y.


### ELECTRICAL ANÆSTHESIA.

BUFFALO, N. Y., Dec. 15, 1858.

JOHN T. TOLAND,—

I sent you a letter a few days ago, requesting you to withdraw my advertisement for the present. And now, I am on this subject, it reminds me that there is a strong prejudice in the Dental Profession averse to patents, which is very absurd until the Profession are pre pared to pay a man for years of toil and outlay. Bah! I have no patience with such twaddle. Just see how the Profession have sustained me. I claim to be the original discoverer of the Electrical Anæsthesia, and applied for a patent, intending to charge Dentists $20 for office rights, and *give* the *surgical* part of it to the world. Another person, it seems, by *some means*, obtained a patent ahead of me. I considered, (by advice of counsel,) that it would be best to withdraw my claim. I did so, as I was not prepared to litigate; it is proved that my process is far superior to the patent, and *yet, although* I have advertised in two Dental Periodicals to sell a Book of Instructions of the *best mode known* of using Electricity in Dental operations, *for One Dollar*, I have *not sold Books enough to pay for the advertisements.* I should like to have some person devise a process by which the discoverer of a new fact in science appertaining to Dental practice, (which *is* really *useful* and *new*,) may have some prospect of being paid a moderate compensation for his outlay of time and money. Honor or Fame is poor substitute for Bread and Fuel, at this season of the year at any rate.

Allow me to suggest, also, that inventors as a class are invariably poor, and such being the case, fame is a poor substitute for what they ought to have.

It appears to me the owner of the patent has fared worse than I have. One reason I suppose is the process does not work well. My advertising has brought a host of letters from all parts, full of frivolous questions, the most of them aiming to obtain the mode of operating by letter, and thereby save the dollar charged for the pamphlet, many of these minus a return stamp. I am sorry to say it, but it really seems to me the Profession will pay for anything that is new and useful if they can't help it. You asked me for a short dissertation on Electrical Anæsthesia! here it is—you may publish it if you please.

I am, sir, with great respect, your friend,

W. G. OLIVER.

